# Effects of copper, and aluminium in ionic, and nanoparticulate form on growth rate and gene expression of *Setaria italica* seedlings

**DOI:** 10.1038/s41598-024-66921-1

**Published:** 2024-07-10

**Authors:** Mateusz Kulasza, Anna Sielska, Magdalena Szenejko, Marianna Soroka, Lidia Skuza

**Affiliations:** 1 Institute of Biology, University of Szczecin, 71415 Szczecin, Poland; 2 Institute of Marine and Environmental Sciences, University of Szczecin, 71412 Szczecin, Poland; 3 Department of Genetics and Genomics, Institute of Biology, University of Szczecin, 71412 Szczecin, Poland; 4 Centre for Molecular Biology and Biotechnology, Institute of Biology, University of Szczecin, 71415 Szczecin, Poland; 5 Doctoral School, University of Szczecin, 70383 Szczecin, Poland

**Keywords:** *Setaria italic*, Nanoparticles, Copper, Aluminium, Nanotoxicology, Gene expression, Ecology

## Abstract

This study aims to determine the effects of copper, copper oxide nanoparticles, aluminium, and aluminium oxide nanoparticles on the growth rate and expression of ACT-1, CDPK, LIP, NFC, P5CR, P5CS, GR, and SiZIP1 genes in five days old seedling of *Setaria italica* ssp. *maxima,* cultivated in hydroponic culture*.* Depending on their concentration (ranging from 0.1 to 1.8 mg L^−1^), all tested substances had both stimulating and inhibiting effects on the growth rate of the seedlings. Copper and copper oxide-NPs had generally a stimulating effect whereas aluminium and aluminium oxide-NPs at first had a positive effect but in higher concentrations they inhibited the growth. Treating the seedlings with 0.4 mg L^−1^ of each tested toxicant was mostly stimulating to the expression of the genes and reduced the differences between the transcript levels of the coleoptiles and roots. Increasing concentrations of the tested substances had both stimulating and inhibiting effects on the expression levels of the genes. The highest expression levels were usually noted at concentrations between 0.4 and 1.0 mg/L of each metal and metal nanoparticle, except for SiZIP1, which had the highest transcript amount at 1.6 mg L^−1^ of Cu^2+^ and at 0.1–0.8 mg L^−1^ of CuO-NPs, and LIP and GR from the seedling treated with Al_2_O_3_-NPs at concentrations of 0.1 and 1.6 mg L^−1^, respectively.

## Introduction

Metal ions and metal nanoparticles (NPs) are widely used in different branches of the industry due to their various physicochemical properties. Because of their extensive use in factories and households, they end up in the environment as efflux and aerosols, as well as waste from the weathering of products containing them, such as clothing, paint, or pipes. Metal ions and metal NPs are present in every environment, dispersed in air, water, underground water, and soil. Plants uptake them with water and minerals and are subjected to their toxic effects^1–3^.

Copper is an essential element for plants and tends to accumulate mostly in roots and chloroplasts^4,5^. Optimal copper concentration in nutrient media, including soil, is 10^−14^ to 10^−16^ M^6^. Cu^2+^ is crucial for proper enzymatic activity of plastocyanin, cytochrome *c*, both taking part in electron transport without which photosynthesis and respiration cannot take place^7^. Lower concentrations may lead to copper deficiency symptoms, which include malformation of the leaves, chlorosis, and necrosis^6^, while excess copper may result in growth inhibition, lower biomass, reduced respiration, and photosynthesis rate, lipid peroxidation and cause chlorotic symptoms and chloroplast damages^8–11^. Copper and copper NPs are widely used in various industries. Common applications of Cu^2+^ and CuO-NPs may include building constructions, electronic products, telecommunication, fertilizers, hearing and visual aid, and other medical devices or in water and waste management systems. CuO-NPs are used in paint, optical and medical instruments, pipes, and conductive materials production. They can also act as drug carriers or be used in bioimaging^1–3,12^.

Al^3+^ and Al_2_O_3_-NPs are also commonly used in the industry, mostly as building materials for components or as adsorptive elements, as well as in the process of removing sulfur from natural gas. Aluminium is the most abundant metal in Earth’s crust and has found its use in pro-duction of cans or vehicle parts^13^, however it is mostly toxic. Because of the fact, that it is easily available for plants, in some cases, it may play a positive role in the growth and development of plants^14–17^. However, plant roots are the most sensitive to the toxic effects of aluminium, and their growth and ability to uptake water and minerals is retarded^14,18^.

The toxic effects of copper may be caused by several factors, such as the induction of reactive oxygen species (ROS) generation and increase free-radical generation, such as ^⋅^OH and ^⋅^O2^−^. These ROS can damage DNA, proteins, and lipids, and alter the structure of nitrogen bases, which might lead to mutations (base pair transitions, DNA breaks and unwinding)^12,19–21^. Aluminium catalyzes the formation of ROS and lipid peroxidation, induces modifications in the cytoskeleton structure, and halts cell proliferation. Roots of plants treated with aluminium are brittle, and their tips are damaged and swollen. Necrosis and chlorosis are developed by leaves from excess concentration of aluminium. Aluminium replace Ca^2+^ ions and other cations that helps to maintain the proper structure of the cell wall, making the wall stiff and less stretchable^14,16,22–27^. Metal NPs can induce toxic response by various mechanisms. Due to their increased size compared to bulk material, they can mechanically damage the membranes and disturb functions of membrane proteins. If they penetrate the plasmalemma, they may induce structural changes to the cytoskeleton components, disturb lysosome functioning and induce ROS generation^28–30^. There are many studies focused on toxicity of heavy metals and metal nanoparticles to plants. It was reported by Lee et al.^31^ that copper nanoparticles at concentrations between 20 and 1000 mg L^−1^ and under controlled laboratory conditions, inhibit the growth of *Phasoleus radiatus*, and *Triticum aestivum*. Growth of *Raphanus sativus* was also retarded by copper oxide NPs^32^. Moreover, it was reported that Al_2_O_3_-NPs exert negative effects on plants through oxidative stress and reduced photosynthetic capacity^33,34^.

*Setaria italica* is an important grain crop cultivated throughout the world. It is grown in regions with poor soil because it is well adapted to grow in the environment with low fertility, high salinity and during drought^35–37^. It is popular in countries with warm climate, and it is placed second in the millet production ranking. *S. italica* is extensively researched for its suitability as a C4 grass model plant. Unlike other C4 plants, it has a small genome of about 490 Mbp which makes it easier to study. The plant also has a small size, fast germination time, short life cycle, is highly resistant to drought, and has a diploid nature (2n = 18). Due to its economic importance, it is crucial to evaluate the potential toxic effects of copper and aluminum and their nanoparticles on *S. italica*^38–43^*.*

The aim of the study was to determine the effects of copper, copper oxide nanoparticles, aluminium, and aluminium oxide nanoparticles on the growth rate and expression of genes, that play crucial role in plants’ growth and development, as well as physiological processes: ACT-1, CDPK, LIP, NFC, P5CR, P5CS, GR, and SiZIP1 genes on plants, by using five days *old Setaria italica* ssp. *maxima* seedlings as a model. The aim of the study was to determine the effects of copper, copper oxide nanoparticles, aluminium, and aluminium oxide nanoparticles on the growth rate and expression of genes, that play crucial role in plants’ growth and development, as well as physiological processes: ACT-1, CDPK, LIP, NFC, P5CR, P5CS, GR, and SiZIP1 genes on plants, by using five days old Setaria italica ssp. maxima seedlings as a model.

## Results

### Effects of metal ions and metal nanoparticles on growth of seedlings

The seedlings of *S. italica* ssp. *maxima* showed statistically significant changes in growth rate under the influence of metal ions and metal nanoparticles. Depending on the concentration of the tested substances, both stimulation and retardation of the growth rate of germ roots or coleoptiles were observed. Germ roots were found to be more sensitive to the metal and metal nanoparticle solutions than coleoptiles.

**Figure 1:**
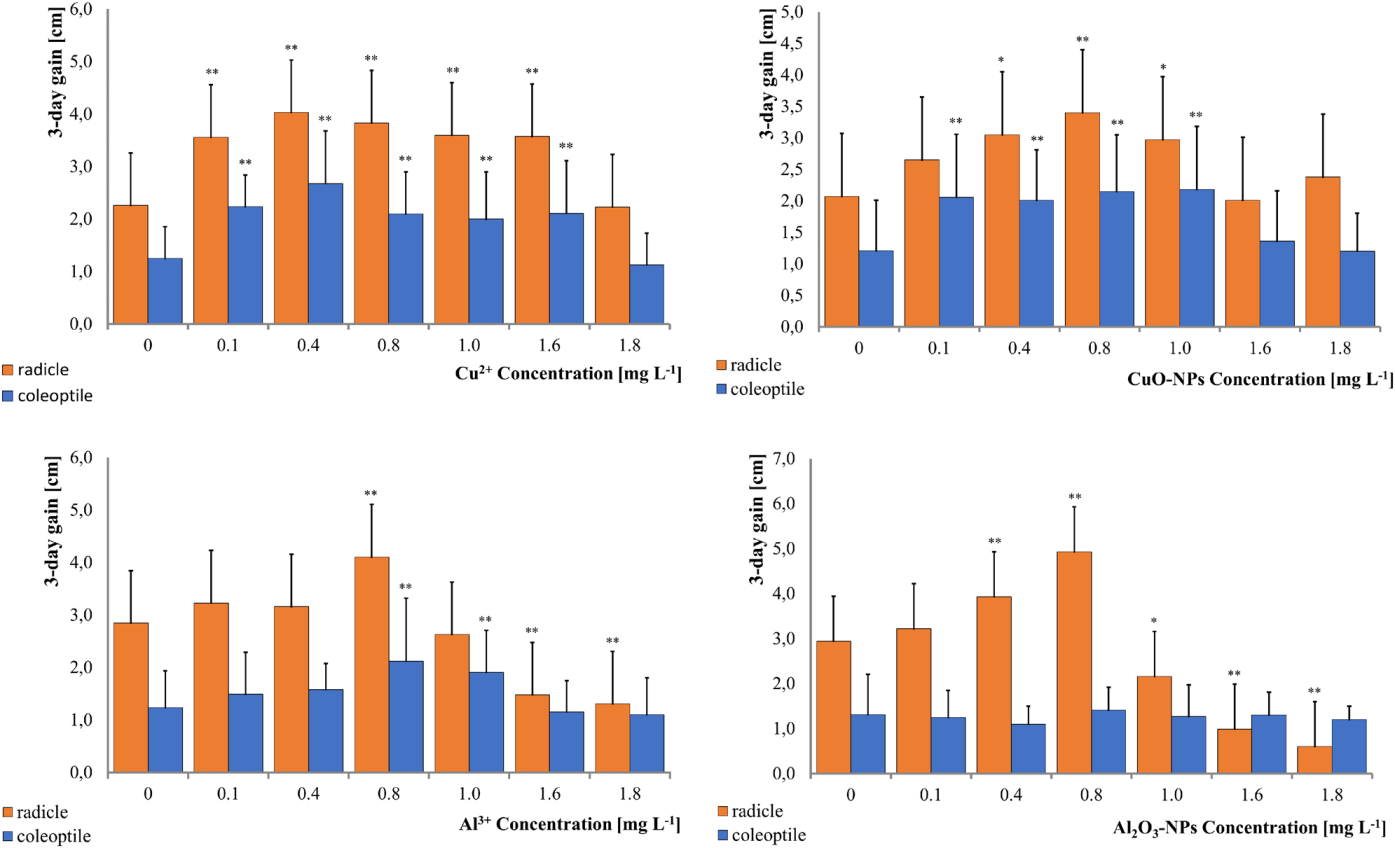
Changes in growth rate of *S. italica* after treatment with Cu (**A**), nano-CuO (**B**), Al (**C**) ND nano-Al_2_O_3_ (**D**). *Dunnett test, statistically significant at p < 0.05; **Dunnett test, statistically significant at p < 0.01*.*

Statistically significant changes in the growth rate of the coleoptiles and germ roots were noted in the broadest range of concentrations (0.1–1.6 mg L^−1^) when seedlings were treated with ionic copper (Table 1) (Fig. 1). The seedlings in the presence of this metal developed germ roots 1.303–1.777 cm longer and coleoptiles 0.754–1.424 cm longer compared to the control. At a concentration of copper of 1.8 mg L^−1^, the growth rate was not significantly different compared to the control. Nanoparticulate CuO-NPs also showed a stimulating effect on the growth rate of *S. italica* seedlings, but within a smaller concentration range (Table 2). The germ roots grew longer when exposed to 0.4–1.0 mg L^−1^, whereas the coleoptiles were the longest with a CuO-NPs concentration of 0.1–1.0 mg L^−1^. At higher concentrations of CuO-NPs, the length of both coleoptiles and germ roots was comparable to the control.

**Table 1:** Mean values and variation of roots and coleoptile length increments of *S. italica* seedlings under conditions of variable concentration of Cu^2+^.

	Cu^2+^ concentration (mg L^−1^)						
	Control	0.1	0.4	0.8	1	1.6	1.8
Root (n = 210) F_6,203_ = 11.062 p = 0.000							
Mean ± SD	2.260 ± 0.9111	3.563** ± 1.2016	4.037** ± 1.1981	3.833** ± 1.2474	3.597** ± 1.2949	3.573** ± 1.5124	2.230 ± 1.0796
Median	2.050	3.550	4.000	3.750	3.750	3.500	2.450
CV	40.31	33.72	29.68	32.54	36.00	42.33	48.41
Range	0.2–4.5	0.5–6.5	2.0–7.0	1.3–6.6	0.0–6.3	0.0–6.5	0.0–4.0
Coleoptile (n = 210) H = 62.068 p = 0.000							
Mean ± SD	1.253 ± 0.6377	2.237** ± 0.6088	2.677** ± 1.0592	2.090** ± 0.7658	2.007** ± 0.9414	2.113** ± 0.9779	1.33 ± 0.6211
Median	1.300	2.050	2.500	2.150	1.900	2.200	1.000
CV	50.88	27.22	39.57	36.67	46.91	46.27	54.80
Range	0.1–2.5	1.2–3.5	0.7–5.3	0.3–3.7	0.3–3.9	0.3–4.5	0.1–3.0

SD—standard deviation; CV—coefficient of variation (%).

*Dunnett’s test, statistically significant at p < 0.05.

**Dunnett’s test, statistically significant at p < 0.01.

**Table 2:** Mean values and variation of roots and coleoptile length increments of *S. italica* seedlings under conditions of variable concentration of CuO-NPs.

	CuO-NPs concentration (mg L^−1^)						
	Control	0.1	0.4	0.8	1	1.6	1.8
Root (n = 210) H = 25.579 p = 0.000							
Mean ± SD	2.077 ± 1.1091	2.657 ± 1.2350	3.050* ± 1.3053	3.430** ± 1.7833	2.970* ± 1.3762	2.017 ± 1.0185	2.377 ± 0.7951
Median	2.000	2.650	3.600	3.500	3.050	2.250	2.350
CV	53.41	46.49	42.80	51.99	46.34	50.50	33.45
Range	0.0–4.3	0.5–4.8	0.0–5.0	0.0–7.1	1.0–5.4	0.0–4.5	1.0–4.4
Coleoptile (n = 210) F_6,203_ = 8.161 p = 0.000							
Mean ± SD	1.213 ± 0.8097	2.067** ± 1.0310	2.010** ± 0.8450	2.150** ± 0.9511	2.183** ± 0.9931	1.363 ± 0.8168	1.203 ± 0.6133
Median	1.250	2.000	2.000	2.100	2.150	1.500	1.350
CV	66.74	49.89	42.04	44.24	45.49	59.92	50.97
Range	0.0–2.5	0.3–4.3	0.2–3.7	0.5–4.7	0.0–4.6	0.0–2.9	0.0–2.2

SD—standard deviation; CV—coefficient of variation (%).

*Dunnett’s test, statistically significant at p < 0.05.

**Dunnett’s test, statistically significant at p < 0.01.

Aluminium had a stimulating effect on the growth rate of *S. italica* seedlings within a concentration rate between 0.1 and 1 mg L^−1^, whereas higher concentrations had an adverse effect and caused the seedlings to grow 50% shorter than the control (Table 3). Coleoptiles also grew longest in the presence of aluminium within the range of concentrations between 0.1 and 1 mg L^−1^; however, they were more resistant to the negative effects of higher concentrations.

**Table 3:** Mean values and variation of roots and coleoptile length increments of *S. italica* seedlings under conditions of variable concentration of Al^3+^.

	Al^3+^ concentration (mg L^−1^)						
	Control	0.1	0.4	0.8	1	1.6	1.8
Root (n = 210) F_6,203_ = 31.115, p = 0.000							
Mean ± SD	2.847 ± 0.9555	3.230 ± 1.1624	3.160 ± 1.1398	4.107** ± 1.1988	2.630 ± 0.7355	1.480** ± 0.8624	1.310** ± 0.6099
Median	2.950	3.050	2.800	4.05	2.450	1.450	1.200
CV	33.56	35.99	36.07	29.19	27.89	58.27	46.56
Range	0.7–4.9	1.2–6.5	1.2–6.5	2.0–6.4	1.5–4.5	0.2–3.0	0.0–2.3
Coleoptile (n = 210) F_6,203_ = 7.005, p = 0.000							
Mean ± SD	1.237 ± 0.7568	1.490 ± 0.8062	1.580 ± 0.5610	2.123** ± 1.1655	1.910** ± 0.8632	1.153 ± 0.6339	1.103 ± 0.7054
Median	1.300	1.500	1.700	2.150	1.600	1.150	1.200
CV	61.20	54.10	3.51	54.89	45.19	54.97	63.93
Range	0.1–2.6	0.0–2.9	0.2–2.5	0.4–4.5	0.5–3.5	0.0–2.7	0.0–2.8

SD—standard deviation; CV—coefficient of 1070 variation (%).

*Dunnett’s test, statistically significant at p < 0.05.

**Dunnett’s test, statistically significant at p < 0.01*.*

Roots were highly sensitive to variable Al_2_O_3_-NPs concentrations (Table 4). Statistically significant stimulation of their length growth was noted in the range of 0.4–0.8 mg L^−1^, up to 2 cm longer than the control sample. Higher concentrations of Al_2_O_3_-NPs had a toxic effect on *S. italica* seedlings, causing significantly smaller increases in radicle length. A concentration of 1.8 mg L^−1^ caused roots to grow 80% shorter (0.603 cm) than the control sample (2.940 cm).

**Table 4:** Mean values and variation of roots and coleoptile length increments of *S. italica* seedlings under conditions of variable concentration of Al_2_O_3_-NPs.

	Al_2_O_3_-NPs (mg L^−1^)						
	Control	0.1	0.4	0.8	1	1.6	1.8
Root (n = 210) H = 141.551 p = 0.000							
Mean ± SD	2.940 ± 1.0702	3.220 ± 1.2772	3.933** ± 1.1912	4.933** ± 1.0558	2.163* ± 1.0562	0.990** ± 0.7854	0.603** ± 0.5288
Median	2.850	3.200	3.950	5.000	2.200	1.000	0.700
CV	36.40	39.67	30.28	21.40	48.82	49.33	87.65
Range	1.1–5.5	1.2–5.5	1.0–5.5	2.5–6.4	0.6–4.8	0.0–3.4	0.0–1.8
Coleoptile (n = 210) H = 5.202 p = 0.518							
Mean ± SD	1.310 ± 0.9353	1.250 ± 0.6791	1.097 ± 0.4902	1.417 ± 0.5093	1.270 ± 0.7387	1.307 ± 0.5889	1.200 ± 0.3869
Median	1.100	1.300	1.050	1.400	1.100	1.200	1.200
CV	71.39	54.33	44.70	35.95	58.16	45.07	32.24
Range	0.2–3.6	0.3–2.6	0.1–2.1	0.3–2.2	0.1–3.0	0.4–2.5	0.5–1.9

SD—standard deviation; CV—coefficient of variation (%).

*Dunnett’s test, statistically significant at p < 0.05.

**Dunnett’s test, statistically significant at p < 0.01.

### Differences in gene expression levels between coleoptiles and roots

**Figure 2:**
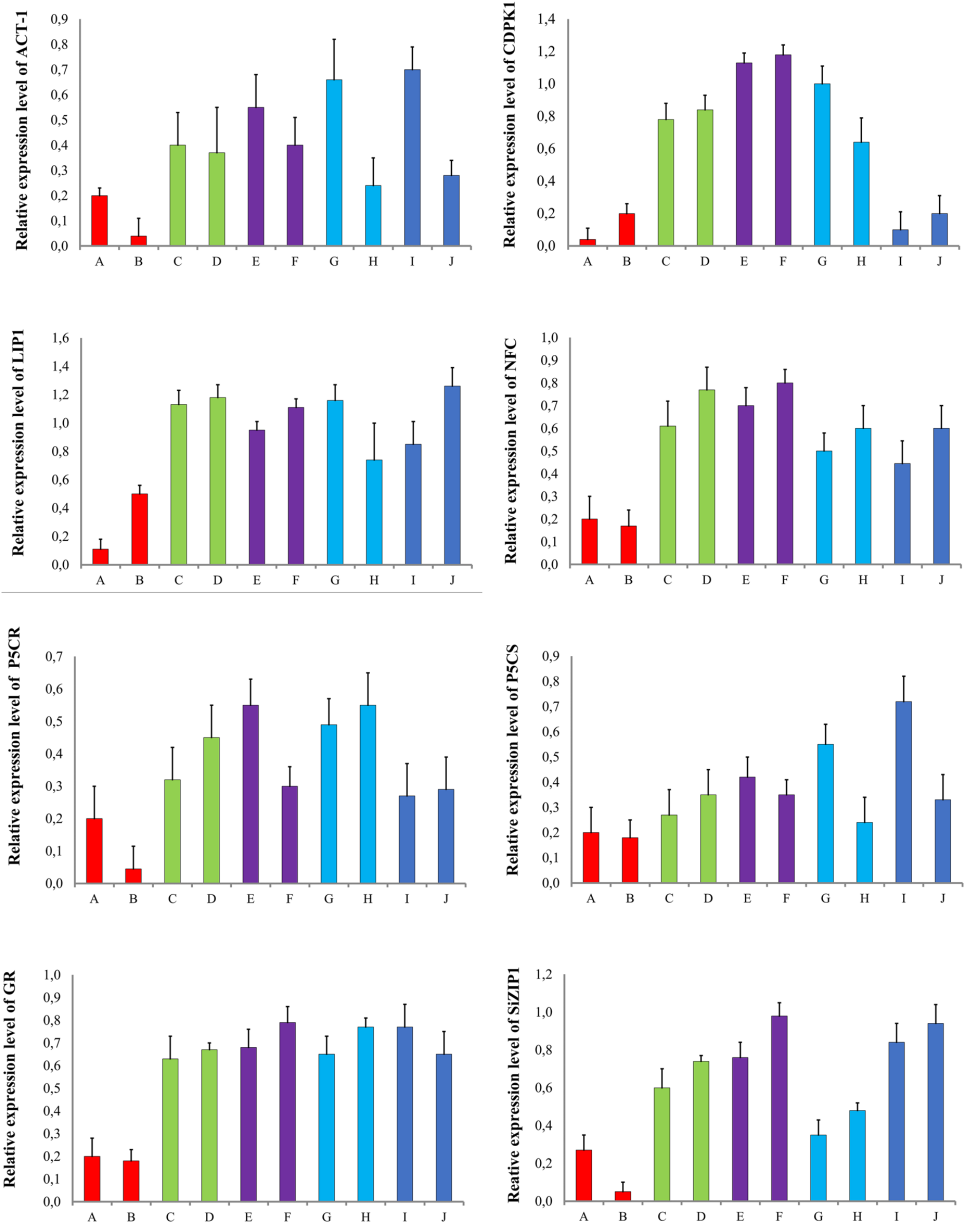
Comparison of relative expression levels of ACT-1, CDPK, LIP, NFC, P5CR, P5CS, GR, SiZIP1 after treatment with 0.4 mg of tested substances. Vertical bars represent SD. (**A**) Control coleoptile, (**B**) control radicle, (**C**) copper coleoptile, (**D**) copper radicle, (**E**) copper oxide NPs coleoptile, (**F**) copper oxide NPs radicle, (**G**) aluminium coleoptile, (**H**) aluminium radicle, (**I**) aluminium oxide NPs coleoptile, (**J**) aluminium oxide NPs radicle.

In control samples expression levels of ACT-1, PCR, and SiZIP were significantly higher in the coleoptiles than in the roots, even around 10-times for SiZIP (Fig. 2). LIP expression was also lower in the roots but only around 2-times, and GR, NFC, and PCS showed only slightly higher level of transcripts in the coleoptiles compared to the roots. Only instance where there was significantly more transcript of this gene in the roots compared to the coleoptiles was with the CDPK gene.

After incubating the samples with the tested toxicants at a concentration of 0.4 mg L^−1^ each, most of them showed a shift in expression levels.

In samples treated with copper and copper oxide nanoparticles from seedlings germinated with Cu, the expression of the ACT-1 gene was comparable in the coleoptiles and roots. The highest expression level of ACT-1 was observed in the coleoptiles treated with CuO-NPs, which was about twice as high as the expression levels in the other samples. In samples treated with aluminium and aluminium oxide-NPs, ACT-1 expression remained significantly higher in coleoptiles than in roots. The highest amount of transcript was detected in the sample germinated with aluminium, around three times higher than in roots treated with the same toxicant.

For CDPK, the transcript level was comparable in the coleoptiles and roots of samples treated with Cu. In samples treated with CuO-NPs, the expression levels of CDPK were also comparable between the coleoptiles and roots, without any significant decrease. As for aluminium, the transcription level was higher in the roots than in the coleoptiles only in the samples treated with Al_2_O_3_-NPs, while in the samples with aluminium, the expression was higher in the coleoptiles. The amount of transcript in the coleoptiles of the sample treated with aluminium was comparable to the amount of transcript from the roots of the sample with Al_2_O_3_-NPs, and the same was observed for the roots and coleoptiles in the two other samples.

LIP expression in the seedlings treated with Cu and CuO-NPs was comparable between all four variants. In the case of aluminium, the expression was still higher in the coleoptiles in both samples treated with Al and Al_2_O_3_-NPs, but the difference was not significant. The higher amount of transcript was detected in the samples germinated with Al^3+^ for both organs.

For NFC, comparable expression levels were observed in the roots and coleoptiles of seedlings germinated with Cu, as well as in the coleoptiles of the sample treated with CuO-NPs. The highest expression was induced by CuO-NPs in the roots, but it was not significantly different from the other three samples. With aluminium treatment, NFC expression was higher in the roots in both cases, but only slightly. The highest amount of transcript was detected in the roots from the samples treated with Al_2_O_3_-NPs, and it was approximately two times higher than in the coleoptiles treated with the same substance. The amount of transcript was comparable between the coleoptiles from both variants and was only slightly lower than in the sample obtained from the roots treated with Al^3+^.

The amount of P5CR transcript was comparable in the coleoptiles and roots of seedlings germinated in the presence of CuO-NPs, as well as in the roots of the sample treated with CuO-NPs. The highest amount was detected in the coleoptiles from the CuO-NPs-treated group, but it was not significantly different from the other three samples. With aluminium, the expression was still induced more in the coleoptiles treated with both Al^3+^ and Al_2_O_3_-NPs, but now the difference compared to the roots was not significant. The expression was comparable between coleoptiles from the samples with Al^3+^ and Al_2_O_3_, as well as between the roots.

With the P5CS gene, a higher amount of transcript was detected in the roots than the coleoptiles of seedlings treated with Cu, whereas in the CuO-NPs-treated seedlings, the highest level was observed in the coleoptiles. With aluminium treatment, a significant difference in expression levels between coleoptiles and roots was detected for both Al^3+^ and Al_2_O_3_-NPs. The gene was more highly expressed in the coleoptiles than in the roots, with the highest amount of transcript found in the coleoptiles treated with Al_2_O_3_-NPs, where it was three times higher than in the roots from both treatment groups. In the group treated with Al^3+^, the expression was around two times higher in the coleoptiles than in the roots.

For the GR gene, expression levels were comparable in both organs of Cu-treated seedlings and in the coleoptiles of CuO-NPs-treated seedlings, with the highest amount of transcript detected in the coleoptiles from the CuO-NPs-treated group. Expression levels were similar in both organs for samples treated with Al^3+^, but higher than in the samples treated with Al_2_O_3_-NPs, although not significantly. In the Al_2_O_3_-NPs-treated group, the amount of transcript was slightly higher in the coleoptiles than in the roots.

Regarding SiZIP, expression levels were comparable between coleoptiles from seedlings germinated with Cu and those from the CuO-NPs treatment, which was also observed in the roots. For aluminium treatment, expression levels were comparable in all samples except for the roots treated with Al^3+^, where it was approximately two times higher.

### Effects of copper oxide nanoparticles on stress genes expression

**Figure 3:**
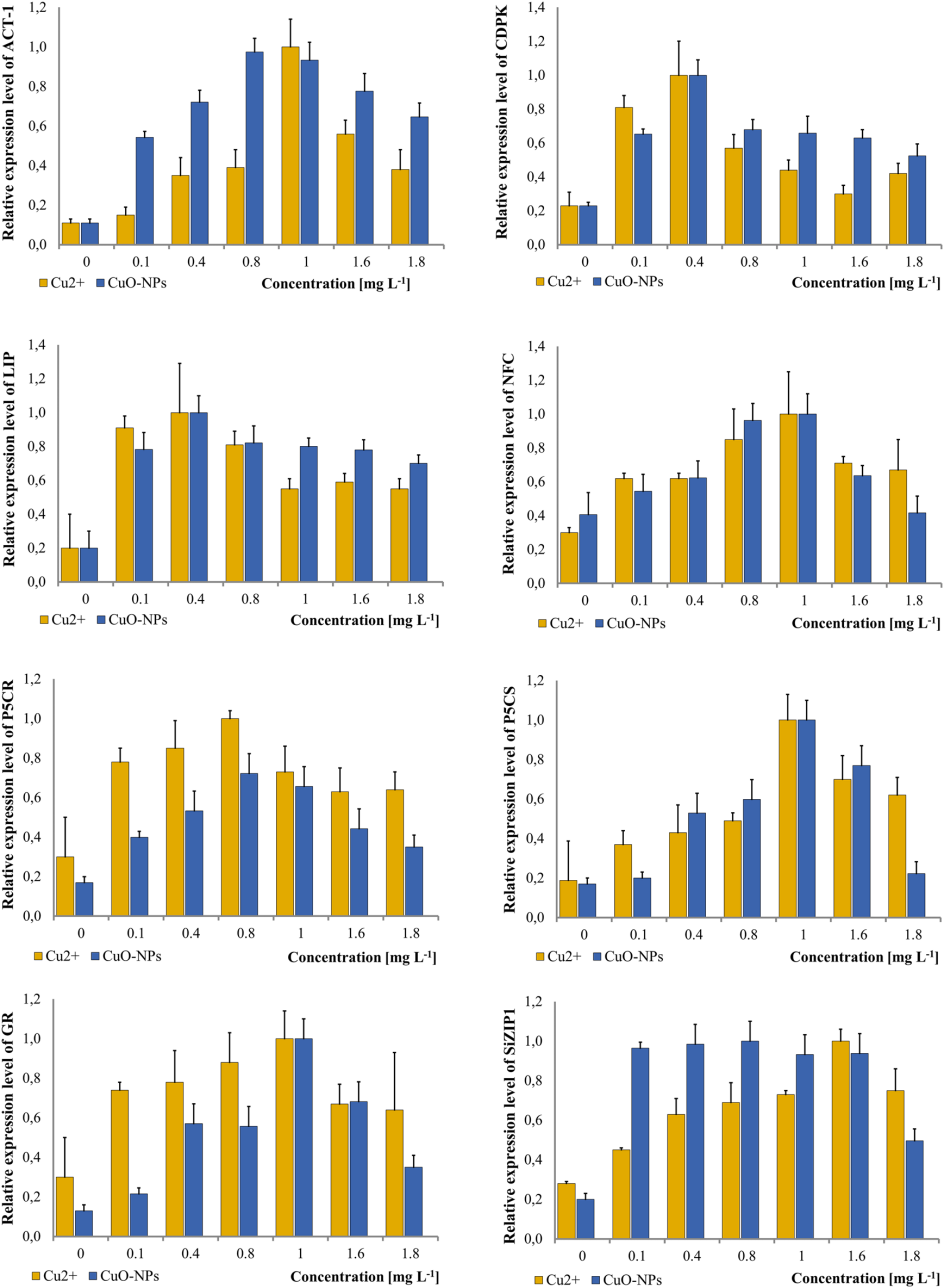
Changes in expression levels of ACT-1, CDPK, LIP, NFC, P5CR, P5CS, GR, SiZIP1 after the treatment with Cu^2+^ and CuO-NPs. Vertical bars represent SD.

The expression level of the ACT-1 gene in seedlings increased gradually from 0 to its highest level at 0.8 mg L^−1^ CuO-NPs, which was around 10 times higher than the control sample (Fig. 3). At a concentration of 1 mg L^−1^ CuO-NPs, the transcript amount started to decrease, and at 1.8 mg L^−1^, it was around 7 times higher than in the samples obtained from seedlings germinated on water only.

For the CDPK gene, the transcript amount increased between the control and the concentration of 0.4 mg L^−1^ CuO-NPs, where it was around 5 times higher. Afterward, it declined and stayed at a relatively constant level of around 3–4 times higher than the control throughout the remaining tested concentrations. LIP expression behaved similarly, with the expression level being fourfold and fivefold higher at concentrations of 0.4 and 0.8 mg L^−1^ CuO-NPs, respectively, compared to the control sample. Then, it stayed at an around 4 times higher level between 0.8 and 1.8 mg L^−1^ CuO-NPs.

For the NFC gene, the expression level increased between the control and 1 mg L^−1^ of CuO-NPs, at which it was around 3-times higher. At the concentration of 1.6 mg L^−1^, the expression started to decrease, and at 1.8 mg L^−1^, it reached a level comparable to the control sample. The expression of the P5CR gene had a similar pattern, but the highest amount of the transcript was detected at 0.8 mg L^−1^ CuO-NPs, after which it started to decrease, with the lowest level being around fourfold higher at 1.8 mg L^−1^ compared to the control sample.

The amount of the transcript of the P5CS gene was comparable between the control and the concentration of 0.1 mg L^−1^, and it started to increase at 0.4 mg L^−1^ of CuO-NPs. At 1 mg L^−1^, it reached the highest amount, which was around fivefold higher than in the samples from seedlings grown in water. At the concentration of 1.8 mg L^−1^, the expression level dropped back to the level detected in the control.

The expression level of the GR gene increased up to 1 mg L^−1^ of CuO-NPs, with two-, four- and tenfold higher expression levels detected at the concentrations of 0.1, 0.4 and 0.8, and 1 mg L^−1^ of CuO-NPs respectively, compared to the control. At 1.8 mg L^−1^, it dropped to a value around twofold higher than the control.

In contrast, the pattern of expression of the SiZIP gene was different from the other genes. It increased starting at a concentration of 0.1 mg L^−1^ CuO-NPs and remained at a fivefold higher level up to a concentration of 1.6 mg L^−1^. A decrease in the expression level was observed in the sample obtained from the seedlings germinated on a solution containing 1.8 mg L^−1^ CuO-NPs, and compared to the control, it was around twofold higher.

### Effects of copper on gene expression

The expression level of the ACT-1 gene increased gradually from 0 to the highest level at 1 mg L^−1^ Cu^2+^ and was approximately tenfold higher than in the control. The transcript level decreased at 1.6–1.8 mg L^−1^ Cu^2+^ (Fig. 3).

For the CDPK gene, the transcript amount was approximately four- and fivefold higher compared to the control for concentrations of 0.1 and 0.4 mg L^−1^, respectively. The transcript level started to decrease at 0.8 mg L^−1^ Cu^2+^ where it was threefold higher compared to the control. At higher concentrations, the level was twofold higher than in the sample obtained from the seedlings germinated on water.

The pattern of expression of the LIP gene was similar to that of CDPK. The highest expression level was observed in the sample obtained from the seedlings treated with 0.4 mg L^−1^ Cu^2+^, reaching values approximately fivefold higher compared to the control.

At the concentration of 0.8 mg L^−1^, the transcript level slightly decreased to a level fourfold higher compared to the control. Then, at 1–1.8 mg L^−1^ Cu^2+^, it remained at a level around threefold higher than in the sample obtained from the seedlings treated with water only.

The expression of the NFC gene was found to increase to approximately three times the level of the control at concentrations of 0.1–0.4 mg L^−1^ Cu^2+^, and four times higher at 0.8 mg L^−1^. The highest amount of transcript was detected in the sample obtained from seedlings treated with 1 mg L^−1^ Cu^2+^. At concentrations of 1.6 and 1.8 mg L^−1^, the amount of transcript showed a similar level of expression to that observed at concentrations of 0.1–0.4 mg L^−1^.

PCR analysis showed that the expression levels of the gene increased progressively with each concentration of Cu^2+^, reaching a maximum at 0.8 mg L^−1^ where it was approximately five times higher than in the control. At concentrations of 1–1.8 mg L^−1^ Cu^2+^, the expression levels were approximately 3–4 times higher compared to the control group.

For the PCS gene, the expression level increased gradually with each concentration of Cu^2+^, starting from a value of approximately two times higher than the control and reaching a maximum of around five times higher at a concentration of 1 mg L^−1^ Cu^2+^. However, the expression level started to decrease in samples treated with 1.6–1.8 mg L^−1^ Cu^2+^. In the sample obtained from seedlings treated with a solution containing 1.8 mg L^−1^ Cu^2^, the expression level was around three times higher than in the control.

The expression level of the GR gene also increased gradually up to 1 mg L^−1^ Cu^2+^ but the difference between the control and the first three concentrations tested was greater than with the previous gene. At 0.8 mg L^−1^, the expression level was three times higher, and at 1 mg L^−1^ Cu^2+^ it was four times higher compared to the control. However, the expression level started to decrease at 1.6–1.8 mg L^−1^ and compared to the sample from seedlings treated with water, it was only around two times higher.

With SiZIP, the amount of transcript increased gradually up to a concentration of 1.6 mg L^−1^ Cu^2+^ where it reached a level approximately three times higher than in the control. However, at 1.8 mg L^−1^, it decreased to a value of around two times higher than in the control.

### Effects of aluminium on gene expression

**Figure 4:**
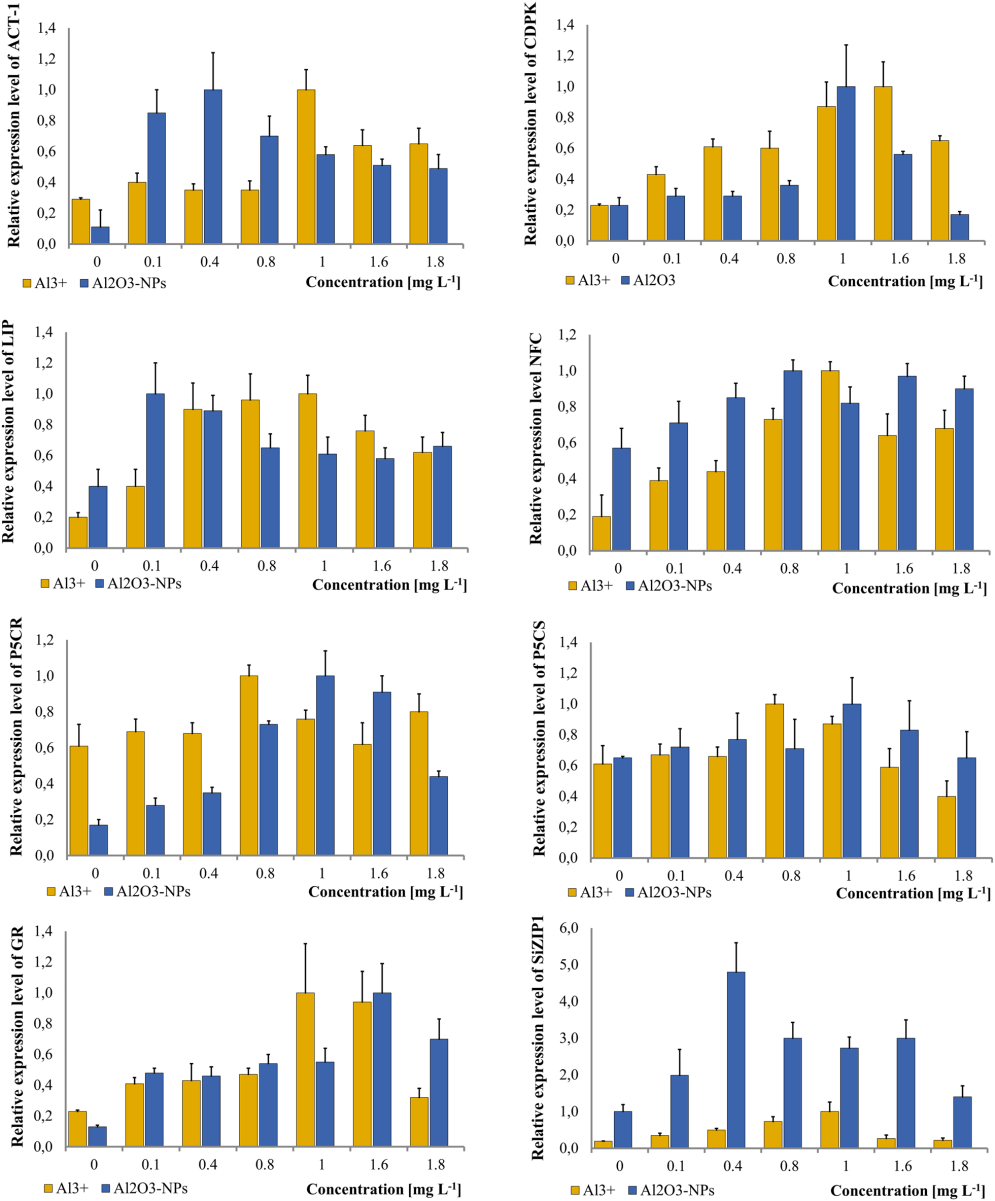
Changes in expression levels of ACT-1, CDPK, LIP, NFC, P5CR, P5CS, GR, SiZIP1 after the treatment with Al^3+^ and Al_2_O_3_-NPs. Vertical bars represent SD.

The relative expression level of the ACT-1 gene showed similar values within the range of 0–0.8 mg L^−1^ of Al^3+^, and the level of expression reached a value about three times higher than the control (Fig. 4). An increase in the expression level occurred at a concentration of 1 mg L^−1^, where it reached a value three times higher than the control. However, in subsequent concentrations, the expression level decreased, and the values were comparable.

The expression levels of CDPK showed a significant increase with increasing concentrations of Al^3+^. At a concentration of 0.1 mg L^−1^, the level of expression was twice as high compared to the control, and at concentrations of 0.4 and 0.6 mg L^−1^, it was three times higher. At a concentration of 1 mg L^−1^, it was four times higher, and at a concentration of 1.6 mg L^−1^, it was also four times higher, followed by a decrease in the expression level with 1.8 mg L^−1^ of Al^3+^.

The expression of LIP also showed significant changes. At a concentration of 0.1 mg L^−1^, the level of expression was around two times higher than the control sample, and at concentrations of 0.4 and 0.8 mg L^−1^ of Al, it was around four times higher. At 1 mg L^−1^ of Al^3+^, it was five times higher compared to the control sample. However, with higher concentrations, the amount of mRNA from the LIP gene decreased.

The expression of the NFC gene followed a somewhat similar pattern as the LIP gene, with concentrations of 0.1 and 0.4 mg L^−1^ inducing around 2-times higher levels of expression. At the concentration of 0.8 mg L^−1^, the expression level was around 4-times higher compared to the control sample, whereas with 1 mg L^−1^ of Al^3+^, it was around 5-times higher. Concentrations of 1.6 and 1.8 mg L^−1^ of Al^3+^ induced repression of the expression, but it was still significantly higher compared to the control.

The amount of the transcript of the P5CR gene did not show significant differences between the different concentrations of Al^3+^. The highest mRNA amount was detected in the seedlings that were germinated in the presence of 1 mg L^−1^ of Al^3+^, but the change was not significantly different compared to the control sample.

The P5CS gene did not exhibit a significant rise in the expression level. The amount of the transcript in the control sample and in the samples with 0.1 and 0.4 mg L^−1^ of Al^3+^ was comparable, and the concentration of 0.8 mg L^−1^ caused only a mild increase in the transcript amount. Higher concentrations of Al^3+^ induced a decrease in the mRNA level, with 1.8 mg L^−1^ of Al being lower compared to the control sample.

In the case of the GR gene, the amount of mRNA gradually increased up to the concentration of 1 mg L^−1^ of Al^3+^, with values around 2 times higher at concentrations of 0.1–0.8 mg L^−1^ compared to the control. At 1 mg L^−1^ of Al^3+^, the amount was around 5 times higher compared to the control, but then it started to decrease at 1.6 mg L^−1^ and became comparable to the control at 1.8 mg L^−1^.

The expression level of the SiZIP gene also gradually increased with increasing concentrations of Al^3+^ up to the concentration of 1 mg L^−1^, where it reached a value five times higher compared to the control sample. In the presence of higher concentrations of aluminium in the medium, the SiZIP expression level decreased to a level comparable to that of the control sample.

### Effects of aluminium oxide nanoparticles on the gene expression

The expression level of the ACT-1 gene increased by approximately fourfold at a concentration of 0.1 mg L^−1^ of Al_2_O_3_-NPs compared to the control (Fig. 4). At a concentration of 0.4 mg L^−1^, the expression level reached a level around five times higher than in the control. From a concentration of 0.8 mg L^−1^, there was a gradual decrease in the gene expression.

The mRNA content of the LIP gene peaked at 0.1 mg L^−1^ of Al_2_O_3_-NPs and was around three times higher than in the control sample. A slight decrease in mRNA content occurred at a concentration of 0.4 mg L^−1^, and then it remained at a comparable level for concentrations higher than 0.4 mg L^−1^.

The amount of the CDPK transcript gradually rose between the control sample and the concentration of 0.8 mg L^−1^ of Al_2_O_3_-NPs and at 1 mg L^−1^ it was significantly higher. At the concentration of 1.6 mg L^−1^ the expression level dropped significantly and stayed low at 1.8 mg L^−1^.

The NFC gene transcript increased gradually between the control sample and the concentration of 0.4 mg L^−1^ of Al_2_O_3_-NPs. In the sample obtained from the seedlings grown in the presence of 0.8 mg L^−1^ of Al_2_O_3_-NPs, the amount of the transcript was the highest, and it was two times higher compared to the control sample. In subsequent concentrations, there was only a slight decrease in the content of the gene transcript detected, and the values remained at a similar level.

The level of expression of the P5CR gene gradually increased at concentrations of 0–0.8 mg L^−1^ and was around five times higher than in the control at a concentration of 1 mg L^−1^. At a concentration of 1.6 mg L^−1^, the level of expression was slightly lower than at a concentration of 1 mg L^−1^, whereas at the concentration of 1.8 mg L^−1^ of Al_2_O_3_-NPs, the decrease was significant.

The content of mRNA for the P5CS gene was at a similar level in the seedlings grown in a medium containing 0–0.8 mg L^−1^ of Al_2_O_3_-NPs. A slight increase in transcript content occurred at a concentration of 1 mg L^−1^, and at higher concentrations, it remained at a level similar to that at the concentrations of 0–0.8 mg L^−1^.

The expression level of the GR gene doubled compared to the expression in the control sample, and it remained at a similar level up to the concentration of 1 mg L^−1^ of Al_2_O_3_-NPs. At a concentration of 1.6 mg L^−1^, there was around a five-fold increase in the expression level than in the control, and then at 1.8 mg L^−1^, it was around four times higher than in the control. In the case of the SiZIP gene expression, its level slightly increased at a concentration of 0.1 mg L^−1^ of Al_2_O_3_-NPs, and at a concentration of 0.4 mg L^−1^, it was around five times higher. However, at concentrations of 0.8–1.6 mg L^−1^, the expression level dropped to a level around three times lower compared to the control sample. At the concentration of 1.8 mg L^−1^, it reached a value similar to the sample from the control group.

## Discussion

Due to the constantly increasing use of metals and metal nanoparticles (NPs) in industries and households, they are being released into the environment in large quantities. The excess metal ions and metal nanoparticles present in the environment pose a threat to living organisms, including aquatic and terrestrial species. Concentrations of metals and their corresponding nanoparticles can vary across the world, for example the concentration of copper across the European Union ranges from 9 to over 65 mg kg in agricultural topsoil European^44^. Moreover, the aluminum concentrations in soils can vary from scarce from 0.0003 to 0.047 g kg (France) to very high from 33.0 to 128.5 g/kg (Japan, China). Barretto et al. and Feng et al. reported that there is limited data regarding the concentrations of metal nanoparticles in the aquatic environment^45–47^.

This study was conducted to investigate the effects of metal ions and metal nanoparticles in relatively small concentrations on *S. italica* ssp. maxima seedlings five days post germination. We tested effects of copper, copper oxide NPs, aluminium, and aluminium oxide NPs on growth rate of coleoptiles and roots, expression of genes involved in abiotic stress response, and differences in expression levels between coleoptiles and roots.

The study showed that all tested toxicants initially had a stimulating effect on the growth of *S. italica* ssp. *maxima* seedlings, followed by a deteriorating effect, depending on the concentration. Copper had a growth-stimulating effect in a broad concentration range of 0.1–1.6 mg L^−1^, for both coleoptiles and roots, with roots being more susceptible to the growth-promoting effect. At a concentration of 1.8 mg ^L−1^, the organs grew shorter but were not shorter than the roots in the control sample. Cook et al. also detected a similar stimulating-then-inhibitory effect on the growth of roots of *Phasoleus vulgaris* at concentrations of 0.5–1.5 μM, and they showed that above the concentration of 5.5 μM, copper inhibited root growth^48^. Jiang et al. were also able to detect a stimulation of root growth in Zea mays using 10–15 M Cu^2+^, but higher concentrations had an inhibitory influence^49^. On the other hand, Aly and Mohamed only observed a negative effect of copper on the growth of both shoots and roots of Zea mays up to 100 μM^50^. In *Triticum aestivum*, 50 ppm Cu^2+^ was able to reduce the length of shoots and roots, while 25 ppm was enough to inhibit growth of *Vigna radiata* roots^9,51^.

CuO-NPs had a narrower positive range of action: 0.4–1.0 mg L^−1^ for roots, and 0.1–1.0 mg L^−1^ for coleoptiles but roots showed a higher degree of growth promotion and sensitivity to CuO-NPs. Concentrations above the stimulating range did not cause the organs to grow significantly shorter compared to the control samples. Most of the studies use significantly higher concentrations of tested substances so direct comparisons are mostly difficult.

*Brassica juncea* showed a decrease in both shoot and root growth while treated with CuO-NPs but the concentrations were higher than used in this study^52^. *Vigna radiata* also was negatively influenced by high concentrations of CuO-NPs and grew shorter than in the control sample with roots being more susceptible to the toxic effects^53^. Copper nanoparticles also inhibited growth of *Phasoleus radiates* and *Triticum aestivum* in an experiment performed by Lee^31^. Other plants that were negatively affected by copper NPs and had stunted growth were *Hordeum vulgare*^54^, Elsholtzia splendens^55^, *Avena sativa* or *Trifolium*^56^.

Some studies reported a stimulating effect of copper nanoparticles on plant growth. 20 ppm of CuNPs increased root and shoot length of *Cajanus cajan*^57^ and root and shoot growth of *Lycopersicum esculentum* also was promoted up to this concentration of Cu_2_O-NPs^58^ whereas *Ipomoea batatas* grew longer roots treated with 25 ppm CuO-NPs^59^.

Aluminium ions had also both stimulating and inhibiting effects on the growth of roots and coleoptiles of *S. italica* ssp. *maxima,* however the concentration range of solutions that had a positive effect was narrower than in the case of Cu^2+^ and CuO-NPs. Both roots and coleoptiles grew longest from the seedlings that were treated with 0.1–1 mg L^−1^ of Al^3+^, however coleoptiles showed higher resistance to the toxic effect of the ions. Higher concentrations of Al^3+^ in the medium reduced the growth of both organs and roots were 50% shorter in the presence of 1.8 mg L^−1^ of Al^3+^.

Other studies also detected dual effects of aluminium on plant growth. Tomioka et al. reported that growth of *Quercus serrata* treated with aluminium up to 5 mM was stimulated in the presence of up to 2.5 mM, whereas higher concentrations tended to decrease it^15^. Al^3+^ at 100 and 300 μM stimulated growth of Coffea arabica^60^. Higher concentrations had a negative impact. For *Camelias sinensis* aluminium proved to have a positive and important influence on the plant’s growth^17,61^, and for *Hancornia speciosa* 300 and 600 μM of Al^3+^ promoted root growth^62^.

In this study, *S. italica* ssp. *maxima* seedlings were also treated with Al_2_O_3_-NPs. The growth of roots was stimulated only in the samples containing 0.4 and 0.8 mg L^−1^ of A_l2_O_3_-NPs, while at higher concentrations, they exhibited a lower growth rate compared to the control, with inhibition up to 80%. In contrast, coleoptiles were more resilient to both stimulating and inhibiting effects of Al_2_O_3_-NPs and did not exhibit significant changes in the growth rate.

Other studies, like those involving CuO-NPs, used higher concentrations of Al_2_O_3_-NPs than in this study, making direct comparisons impossible. Growth of *Arabidopsis thaliana* was stimulated by Al_2_O_3_-NPs, even up to 4000 mg L^−1^, which is a considerably higher concentration than those used in this study^63^. *Nigella arvensis* was more sensitive to Al_2_O_3_-NPs, with growth stimulation occurring up to a concentration of 100 mg L^−1^, beyond which it was toxic^64^. *Nicotiana tabacum* exposed to Al_2_O_3_-NPs in concentrations up to 1% exhibited longer roots compared to the control^65^.

It is commonly known that metal nanoparticles, such as CuO-NPs and Al_2_O_3_-NPs can induce alterations in gene expressions^66^. Changes in the levels of expression of eight different genes was also tested when subjecting *S. italica* ssp. *maxima* seedlings to Cu^2+^, CuO-NPs, Al^3+^, and Al_2_O_3_-NPs. Study showed that all the tested metals and metal-NPs had a stimulatory effect to the tested genes ACT-1, CDPK, LIP, NFC, P5CR, P5CS, GR, SiZIP1) up to a certain concentration after which the expression levels started to decline. Other papers that studied the expression of the same genes or with similar functions tend to use plants subjected to different stressors than metal ions, and metal NPs (with some exceptions) so it is difficult to compare out study with them.

The ACT-1 gene belong to a family of histone acetyltransferases (HATs) that transfer acetyl group from acetyl coenzyme A (Ac-CoA) to histones^67^. Acetylation of lysine in histones destabilizes interactions between histones and DNA because acetylated chain loses its positive charge and thus its ability to form salt bridges with negatively charged DNA chain^68^. This process leads to a relaxation of the chromatin structure, so that transcription factors have easier access to target genes^69,70^. Acetylation of histones plays an important role in the stress response in plants by making the genes stimulated by stress inducing factors more accessible for transcription factors^71,72^.

In *S. italica* ssp. *maxima* in the control sample ACT-1 expression was significantly higher in the coleoptiles. After the treatment with 0.4 mg L^−1^ of Cu^2+^ and, and CuO-NPs the amount of transcript was similar in both radicles and coleoptiles apart from the coleoptiles from the CuO-NPs-treated seedlings, where it was higher than in the other samples. When treated with 0.4 mg L^−1^ of Al^3+^, and Al_2_O_3_-NPs the expression level of ACT-1 was still higher in the coleoptiles. The highest levels of expression were detected for the concentrations of 1, 0.8, 1, and 0.4 mg L^−1^ consequently for Cu^2+^, CuO-NPs, Al^3+^, and Al_2_O_3_-NPs, thus metal ions stimulated the highest expression levels in higher concentrations than metal NPs.

In another study performed on *S. italica* drought had a stimulating effects on the expression of ACT-1^73^. Imran et al.^74^ profiled *Gossypium* for the expression patterns of different HATs after treatments with cadmium, zinc, salinity, and drought. The effects were different for different genes, with some of them being up-regulated and some being down-regulated, and expression was mostly detected in the roots, but other organs expressed the genes as well^75^.

The CDPK, calcium-dependent protein kinase, belongs to the family of serine-threonine protein kinases that detect and decode fluctuations of calcium ions in the cytosol which activate their catalytic activity. CDPKs are involved in various physiological processes, also in the defense against abiotic stress^76–78^. In *S. italica* CDPKs play an important role in the defense against desiccation stress, however it depends on the type of the protein, because some genes encoding different CDPKs are under-expressed and some over-expressed during drought^79^.

For CDPK, transcript levels were comparable in coleoptiles and roots of Cu^2+−^ treated samples. In seedlings incubated with nano-CuO, CDPK expression levels were also comparable between coleoptiles and roots. In the case of seedlings germinating in solutions containing Al^3+^ and Al_2_O_3_-NPs, the expression level was higher in the roots than in coleoptiles only in samples treated with Al_2_O_3_-NPs, while in the samples with Al^3+^ the expression was higher in the coleoptile.

In this study the highest amounts of transcript were detected in the samples treated with 0.4 mg L^−1^ of Cu^2+^, and CuO-NPs, and 1 mg L^−1^ for Al^3+^, and Al_2_O_3_-NPs, thus copper and copper oxide NPs had a stimulating effect in a lower concentration.

In *Triticum aestivum*, up-regulation genes encoding CDPKs was detected after exposure to drought and high salinity^80^, while cold treatment, dehydration, and high salinity induced the up-regulation of CDPK expression in Oryza sativa^81,82^. *Vitis amurensis* was characterized by elevated expression level of CDPKs genes after being subjected to osmotic stress and cold treatment^83^, and in the case of *Zea mays* ABA and H_2_O_2_ had that effect^84^. Inserting a gene encoding one of the CDPKs from ginger to *Nicotiana tabacum* improved the latest plant’s resistance to salinity and drought^85^. Ectopic expression of CDPK from apple to *Nicotiana benthiama* increased the resistance of the tobacco plants to salt and cold stress^86^.

LIP gene is a part of a family of early light-induced proteins (ELIPs). ELIPs belong to a polygenic family of light-absorbing complexes that bind chlorophyll and absorb solar energy. These proteins are encoded in the nuclear genome and are post-translationally directed to chloroplasts and bind to the thylakoid membrane. ELIPs accumulate in parts of plants that are exposed to high-intensity light, but their genes can also be transcribed in etiolated organs or those lacking chloroplasts. Light is one of many factors that induce the formation of ROS. ELIPs can perform defensive functions against light damage and other kinds of ROS-inducing stressors. It is postulated that ELIPs prevent the formation of ROS or act as absorbers of excessive excitation energy^87–91^. Apart from intense UV radiation and cold temperature, high salt concentrations in the growth medium, drought, and ABA can up-regulate the expression of ELIPs as it was shown using *Medicago sativa*^92,93^. It is also known that unique resistance of resurrection plants to desiccation is partly due to ELIPs’ protective role^94^.

In this study, LIP expression was around two times higher in the coleoptiles than in the roots in control samples. After treatment with all the metals, and nanometals at the concentration of 0.4 mg L^−1^ expression was still higher in the coleoptiles. Other studies focus mostly on leaves because the proteins coded by ELIPs genes are engaged in the defense against light stress^87–90,92,95^. Although in our study on *S. italica* ssp. maxima the transcript for LIP was detected in the roots it does not mean that a functioning protein is active there so additional test, mostly proteome studies, are to be conducted.

LIP’s expression level was the highest at the same concentration of 0.4 mg L^−1^ when the seedlings were exposed to Cu^2+^ and CuO-NPs. The expression had the highest level at the lowest concentration of 0.1 mg L^−1^ in the case of Al_2_O_3_-NPs. In the case of aluminium expression reached its highest level at the highest concentration of all the treatment which was 1 mg L^−1^. *Hordeum vulgare* was shown to have between five- to eight-fold higher content of mRNA for ELIPs after being exposed to strong light, and a treatment with low temperature at the same time intensified the expression further. After a week under harsh light and cold the levels of ELIPs mRNA were 40-times higher than in the control sample^54^.

NFC or nucleosome assembly factor, on the other hand, belongs to a well conserved family of nucleosome assembly factor NAP) that serve as histone chaperones that facilitate the nucleosome assembly and remodeling of chromatin both under physiological condition like in cell cycle regulation and under stress conditions in which they maintain a genome stability after DNA damage caused by stressors^96–98^. According to Zhu et al.^99^ histone chaperones are necessary for post-embryonic root development, whereas Barna et al.^117^ describe histone chaperones as an important factor in coordination of plant growth, development rate, and age-related pathogen resistance.

The *S. italica* ssp. *maxima* protein had almost the same level of expression in both the coleoptiles and the roots in the control group. After treatment with 0.4 mg L^−1^ of the tested substances, the expression increased in the radicles of the seedling treated with Cu^2+^ CuO-NPs. The expression pattern had behaved similarly after treatment with Al^3+^, and Al_2_O_3_-NPs, where Al^3+^ had stimulated significantly higher expression levels in the roots than in the coleoptiles, and Al_2_O_3_-NPs had stimulated significantly higher expression in the roots. According to other studies, genes encoding chromatin remodeling proteins are expressed in all plant tissues^99–103^.

In this study, the expression levels of NFC in seedlings treated with Cu^2+^, CuO-NPs, and Al^3+^ at the concentration of 1 mg L^−1^ were elevated for all treatments. With Al_2_O_3_-NPs, it was 0.8 mg L^−1^. In a study performed on *S. italica* subjected to drought, NFC also showed elevated levels of expression^73^.

Other genes tested in this study were two genes involved in metabolism of proline, P5CR (Δ-1-pyrroline-5-carboxylate reductase) and P5CS (Δ-1-pyrroline-5-carboxylate synthetase). The first enzyme converts glutamic acid into a reaction intermediate, pyrrolin-5-carboxylate (P5C), which then becomes a substrate for P5CR to produce proline in the cytoplasm and plastids. Proline catabolism takes place in the mitochondria, where it is delivered by active transport. In plants not subjected to stress stimuli, high concentration of proline has a negative effect on their growth and can also lead to cell death. However, when plants are subjected to stress, the toxicity of proline decreases, and it is used in defense processes. Proline under physiological conditions is an important cellular component—it is a component of the cell wall, participated in cell elongation, root hair development, synthesis, and remodeling of the cell wall during growth, as well as seed germination and seedling growth stimulation^104–109^.

P5CR of *Vitis yeshanensis* expressed in *Arabidopsis thaliana* exposed to drought, the transcript was more abundant in the roots^107^. In *Triticum aestivum* stem was characterized by the lowest amount of P5CR transcript compared to radicles, flowers, and leaves^108^. P5CS was expressed in all organs except roots in *Arabidopsis thaliana* in the control group, but after exposition to drought it started to be expressed in these organs as well^110^. Another study also reported the expression in all the organs, including roots^111^. Sorghum expressed P5CS in all tissues in the control group but after subjecting it to high salinity, the transcript had the highest amount in the roots^112^.

Both P5CR and P5CS in this study had a higher expression level in the coleoptiles in the control sample. After treatment with 0.4 mg L^−1^ of Cu^2+^ and CuO-NPs, the expression pattern behaved similarly for both genes—with CuO-NPs it was still higher in the coleoptiles, whereas with Cu^2+^ it started to be expressed more in the radicles. The highest expression from all the treatment was noted after treatment with CuO-NPs. After the treatment with Al^3+^, and Al_2_O_3_-NPs both genes were expressed more in the coleoptiles.

P5CR and P5CS reached the highest levels of expression at the same concentration of 0.8 mg L^−1^ in the samples treated with Al^3+^, and at 1 mg L^−1^ in the sampled from the seedlings subjected to a treatment with Al_2_O_3_-NPs. For Cu^2+^ and CuO-NPs the highest levels of the transcript of both genes were the same for P5CR at the concentration of 0.8 mg L^−1^—for both treatments, whereas for P5CS the concentration was 1 mg L^−1^ for both treatments.

After exposing *Triticum aestivum* to cadmium and copper, the amount of the transcript for P5CS was elevated^113^. On the other hand, in a study performed on *Solanum lycopersicum* both up-regulation and down-regulation of the gene was noted depending on the concentration. 20 ppm of copper, 10–20 ppm of lead, and 10 ppm of cadmium had a stimulating effect on the expression of P5CS, whereas 50 ppm, 50 ppm, and 20–50 ppm of the respective elements had an adverse effect^114^.

Glutathione reductase (GR) belongs to the NADPH-dependent family of oxidoreductases. It is instrumental in protecting cells from ROS by providing a constant GSH pool by catalyzing the GSSG reduction reaction with simultaneous NADPH oxidation, with a molar ratio of NADPH:GSSG being 1 to carry out the reduction of GSSG to GSH. GR forms a homodimer bound by FAD and in most plants has a mass between 100 and 150 kDa and binds FAD to monomer^115–117^.

In *S. italica* ssp. *maxima* in the control group GR gene was only slightly more expressed in the coleoptiles. When treated with 0.4 mg L^−1^ of Cu^2+^ and CuO-NPs the expression pattern shifted to be slightly higher in the roots and the highest transcript amount was detected in the roots from seedlings treated with CuO-NPs. When the seedlings were subjected to a treatment with Al^3+^, and Al_2_O_3_-NPs the expression was at the same level in the coleoptiles and the roots. Al^3+^ additionally stimulated the expression more than Al_2_O_3_-NPs.

In this study, the highest level of expression of GR gene was reached at 1 mg L^−1^ of Al^3+^, Cu^2+^, and CuO-NPs, whereas for Al_2_O_3_-NPs it was 1.6 mg L^−1^. Only in the sample treated with aluminium the lowest level of expression at 1.8 mg L^−1^ was comparable with the expression in the control sample.

In *Arabidopsis thaliana* treatment with copper significantly up regulated the expression of GR^118^ and overexpression of that gene decreased the plant’s sensitivity to toxic action of aluminium^119^, whereas in *Brassica juncea* it made the plant less sensitive to cadmium^120^. Expression level of GR was also elevated by chilling and drought in *Pisum sativa*^121^ as well as in *Oryza sativa* in which it was also elevated by high salinity of the growth medium^122^. Additionally, fumigation and paraquat treatment stimulated the expression of the cytosolic GR gene in drought-resistant and drought-sensitive *Vigna unguiculata*^123^.

Zinc-iron permeases (ZIP) are mainly involved in the transport of zinc and iron; however, their expression may be increased by other metal ions and stressors. Most ZIPs have eight transmembrane domains, with the amino- and carboxyl terminal ends located outside the cell membrane. They contain between 309 and 476 amino acids; the difference in lengths is mainly due to the distance between transmembrane units III and IV being variable domain. In most cases, the variable region is predicted to contain subunits rich in histidyl residues that bind metals^124–126^.

In this study, expression of SiZIP1 in the control sample was significantly higher in the coleoptiles than in the roots, which was similar in *Zea mays* under normal conditions for all ZIPs but one, which had similar expression levels in both shoots and roots^127^. It was also detected in *Arabidopsis thaliana* under normal conditions expression of AtZIP1 is more prominent in the shoots, however, AtZIP2 was expressed similarly in roots and shoots^126^. In the study performed by Burleigh et al. 154 the expression of ZIP in *Medicago truncatula* was expressed similarly in shoots and roots.

After treating *S. italica* ssp. maxima with 0.4 mg L^−1^ of each tested metals, and nanoparticulate metals changes in the expression patterns were noted. Treatment with Cu^2+^ and CuO-NPs stimulated the expression in the radicles to a level higher than in the coleoptiles and it had the highest level in the sample from the seedlings germinated on medium containing CuO-NPs. When seedlings were subjected to a treatment with Al^3+^ and Al_2_O_3_-NPs the amount of transcript for SiZIP1 was similar in all tested samples but in Al-treated plants’ coleoptiles which was significantly higher. On the other hand, Miler et al.^126^ detected higher expression level of AtZIP1 in the shoots of Arabidopsis thaliana treated with Cu^2+^, but AtZIP2 was more expressed in the roots after the same treatment. AtZIP1 had also similar expression levels in shoots and roots after treatment with iron, higher expression level in shoots after treatment with manganese, and higher expression level in roots after manganese, and copper treatment while iron, and zinc induced similar expression in both organs.

*Setaria italica* had the highest expression levels of SiZIP1 at 1 mg L^−1^ when the seedlings were germinated on the medium containing aluminium. For the samples germinated on the medium containing. For the sample treated with Cu^2+^ and Al_2_O_3_-NPs the highest amount of transcript was detected at 1.6 mg L^−1^. Only after treating the plants with CuO-NPs there was no distinctive peak in the transcript levels which stayed significantly higher than in the control samples between the concentrations of 0.1–1.6 mg L^−1^. In the study performed on *Raphanus sativus* root, treatment with cadmium up regulated the expression of ZIP^128^ and vanadium stress had this effect on ZIP expression in *Oryza sativa* roots^129^.Expression of ZIP genes is mostly studied under Zn- and Fe-starvation stress. Zn-starvation stimulates the expression of ZIP in *Oryza sativa*^129^, and *Triticum turgidum*^130^, whereas in *Zea mays* five ZIP genes were up regulated in roots, and one in both roots and shoots.

Cu^2+^ had the highest range of concentrations that stimulated the growth of *S. italica* coleoptiles and radicles. CuO-NPs stimulated the growth of the roots more and in a wider range of concentrations than the coleoptiles. Al^3+^ had a stimulating influence at first but at the highest concentrations it inhibited the growth of the seedlings. Al_2_O_3_-NPs also had at first a stimulating effect on the growth rate of the radicles and at the highest concentrations it inhibited the process, it did not have any effect on coleoptiles though.

Cu^2+^ and CuO-NPs stimulated the expression of the genes in coleoptiles and radicles at the concentration 0.4 mg L^−1^ more than Al^3+^ and Al_2_O_3_-NPs and the differences between the levels of the expression between radicles and coleoptiles were lower. The concentrations of all the tested toxicants that stimulated the expression of the genes were between 0.1 and 1 mg L^−1^ except SiZIP1that had the highest amount of transcript detected with the concentration of 1.6 Cu^2+^ and with CuO-NPs between 0.1 and 0.8 mg L^−1^, and LIP as well as GR in the seedlings treated with 0.1 and 1.6 mg L^−1^ respectively.

In conclusion, the tested metals and metal nanoparticles in the conditions of the study had both stimulating and inhibiting influences on the growth rate and gene expression of *S. italica* ssp. *maxima* seedlings which depends on the concentration of the toxicant.

## Materials and methods

### Plant material and growth conditions

The study was performed on *S. italica* seedlings germinated from seeds, which were incubated in each tested solution and in distilled water as a control sample. Seeds were purchased from a seed shop (ORGANICseeds; product code: 12374-1). Seeds' species was identified and confirmed by a producer. The tested metals and nanoparticulate metals, including copper (266086; powder; particle size < 425 μm), nanoparticulate copper oxide (544868; nanopowder; particle size < 50 nm (TEM)), aluminium (5188573; granular; particle size < 1 mm), and nanoparticulate aluminium oxide (544833; nanopowder; particle size < 50 nm (TEM)), were purchased from Sigma Aldrich and used to prepare solutions (sources of CuO and Al_2_O_3_ nanoparticles, Cu^2+^ and Al^3+^ ions) ranging from 0.1 to 1.8 mg L^−1^ of each tested substance. The solutions were sonicated for 30 min at 30 °C. The seeds were germinated in darkness at 35 °C for 24 h in sterile Petri dishes lined with two layers of sterile absorbent paper.

### Plant length measurements

After the growth period (5 days), *S. italica* seedlings were exposed to natural light, and each seedling was measured with an accuracy of 0.1 cm.

### RNA extraction and reverse transcription

After five days, the seedlings were harvested, ground in pestles with liquid nitrogen, and used to extract RNA with the EXTRACTME Total RNA Kit (Blirt). To detect differences in expression between the coleoptiles and roots, parts of the seedlings from the control sample and from tested samples of 0.4 L^−1^ of each tested toxicant were cut, and RNA was extracted separately. The isolated RNA was evaluated for purity and concentration using a NanoDrop spectrophotometer and used to synthesize cDNA with the RevertAid First Strand cDNA Synthesis Kit (Thermofisher Scientific). The purity and concentration of cDNA were also evaluated using a NanoDrop spectrophotometer.

Obtained cDNA was used to conduct Real-Time PCR to establish the initial transcripts of tested genes. Reaction mix consisted of 0.2 μL of Forward and Reverse primers, 1 μL EvaGreen Polymerase Mix, 10 ng cDNA (1 μL) and 2.4 μl H_2_O. Reactions were performed with primers listed in Table 1. As reference genes for alfa-tubulin and beta-actin were used, because according to the literature, they show the highest expression level stability, when exposed to various environmental changes^131–134^.

### Quantitative PCR

The temperature profile was set to 95 °C for 15 min for polymerase activation, 95 °C for 0.5 min, and the annealing temperature was set differently for each pair of primers for 0.5 min (Table 1). The extension step was set at 72 °C for 1 min and repeated 34 times. To determine the efficiency of the reference genes, qPCR was performed with a set of cDNA dilutions from the control sample (0.01, 0.1, 1, 100, 1000 ng/μL). The results were analysed using CFX Maestro (Bio-Rad) software using ΔΔCq and presented as bar graphs.

### Statistical analysis

The growth rate of *S. italica* was expressed by the average increments of roots and coleoptiles. Mean values of variables and their dispersion were determined. The value of the increments was calculated by subtracting the values on the first day (second day of the experiment) from the last day of measurements (fifth day after seed sowing). The experiment was conducted using a control sample (n = 4). In total, 840 seedlings were evaluated, 30 for each sample. For each combination, compliance with the normal distribution (Shapiro–Wilk test) was tested. The level of significance of the variation in the values of the measurements was assessed based on the analysis of univariate variance (F, at α = 0.05), and in the absence of convergence of data with normal distribution, a non-parametric Kruskal–Wallis (H, at α = 0.05) was used. The obtained results were compared to control samples, and the significance of differences between them was examined using the Dunnett post-hoc test, with significance levels of 0.05 and 0.01. Calculations were performed using Statistica v.13.3.0 software (StatSoft, Inc., 2013).

### Ethical statement

The plant collection and use were in accordance with all the relevant guidelines.

### Supplementary Information

Supplementary Information.

## Data Availability

The datasets generated and analyzed during the current study are available from the corresponding author on reasonable request.

## References

[1] Wuana RA, Okieimen FE (2011). Heavy metals in contaminated soils: A review of sources, chemistry, risks and best available strategies for remediation. ISRN Ecol..

[2] Koivisto AJ (2017). Quantitative material releases from products and articles containing manufactured nanomaterials: Towards a release library. NanoImpact..

[3] Auffan, M. *et al. Towards a Definition of Inorganic Nanoparticles from an Environmental, Health and Safety Perspective*. vol. 4 634–641 (Nature Publishing Group, 2009). 10.1038/nnano.2009.242 19809453

[4] Aguirre, G. & Pilon, M. Copper delivery to chloroplast proteins and its regulation. *Front. Plant Sci*. **6**, (2015). 10.3389/fpls.2015.01250 PMC4709454 26793223

[5] Lin J, Jiang W, Liu D (2003). Accumulation of copper by roots, hypocotyls, cotyledons and leaves of sunflower (Helianthus annuus L.). Bioresour. Technol..

[6] Yruela I (2005). Copper in plants. Braz. J. Plant Physiol..

[7] Ravet K, Pilon M (2013). Copper and iron homeostasis in plants: The challenges of oxidative stress. Antioxid. Redox. Signal.

[8] Marschner, P. Marschner’s Mineral Nutrition of Higher Plants: Third Edition. *Marschner’s Mineral Nutrition of Higher Plants: 3rd Edition* 1–651 (2011) 10.1016/C2009-0-63043-9.

[9] Ciscato M, Valcke R, Loven K, Clijsters H, Navari-Izzo F (1997). Effects of in vivo copper treatment on the photosynthetic apparatus of two Triticum durum cultivars with different stress sensitivity. Physiol. Plant.

[10] Pätsikkä E, Aro EM, Tyystjärvi E (2001). Mechanism of copper-enhanced photoinhibition in thylakoid membranes. Physiol. Plant.

[11] Hafeez, A. *et al.* Effect of heavy metals on growth, physiological and biochemical responses of plants. *Plants and their Interaction to Environmental Pollution: Damage Detection, Adaptation, Tolerance, Physiological and Molecular Responses* 139–159 (2023) 10.1016/B978-0-323-99978-6.00006-6.

[12] Cervantes-Cervantes MP, Calderón-Salinas JV, Albores A, Muñoz-Sánchez JL (2005). Copper increases the damage to DNA and proteins caused by reactive oxygen species. Biol. Trace Elem. Res..

[13] Brough, D. & Jouhara, H. The aluminium industry: A review on state-of-the-art technologies, environmental impacts and possibilities for waste heat recovery. *Int. J. Thermofluids*. **1–2**, (2020).

[14] Bojórquez-Quintal E, Escalante-Magaña C, Echevarría-Machado I, Martínez-Estévez M (2017). Aluminum, a friend or foe of higher plants in acid soils. Front. Plant Sci..

[15] Tomioka R, Oda A, Takenaka C (2005). Root growth enhancement by rhizospheric aluminum treatment in Quercus serrata Thunb. seedlings. J. Forest Res..

[16] Ghanati F, Morita A, Yokota H (2005). Effects of aluminum on the growth of tea plant and activation of antioxidant system. Plant Soil..

[17] Hajiboland, R., Bastani, S., Bahrami-Rad, S. & Poschenrieder, C. Interactions between aluminum and boron in tea (Camellia sinensis) plants. *Acta Physiol Plant* **37**, (2015).

[18] Shi J (2024). Physiological mechanism through which Al toxicity inhibits peanut root growth. Plants.

[19] Rodriguez-Fernandez, M., Iii, F. J. D., Kreutz, C. & Timmer, J. Oxidative Stress, Protein Damage. *Encyclopedia of Systems Biology* 1619–1620 (2013) 10.1007/978-1-4419-9863-7_650.

[20] Salehi F, Behboudi H, Kavoosi G, Ardestani SK (2018). Oxidative DNA damage induced by ROS-modulating agents with the ability to target DNA: A comparison of the biological characteristics of citrus pectin and apple pectin. Sci. Rep..

[21] Rossman TG (1981). Effect of metals on mutagenesis and DNA repair. Environ. Health Perspect..

[22] Pintro, J. C. & Taylor, G. J. Effects of aluminum toxicity on wheat plants cultivated under conditions of varying ionic strength. 10.1081/PLN-120030678. **27**, 907–919 (2007).

[23] Rahman R, Upadhyaya H (2020). Aluminium toxicity and its tolerance in plant: A review. J. Plant Biol..

[24] Mazzuca D, Russo N, Toscano M, Grand A (2006). On the interaction of bare and hydrated aluminum ion with nucleic acid bases (U, T, C, A, G) and monophosphate nucleotides (UMP, dTMP, dCMP, dAMP, dGMP). J. Phys. Chem. B.

[25] Ribeiro MAQ (2013). Aluminum effecgts on growth, photosynthesis, and mineral nutrition of cacao genotypes. J. Plant Nutr..

[26] Simon, L., Smalley, T. J., Jones, J. B. & Lasseigne, L. Aluminum toxicity in tomato. Part 1. Growth and mineral nutrition. **17**, 293–306. 10.1080/01904169409364728. (2008).

[27] Yanlk, F. & Vardar, F. Toxic effects of aluminum oxide (Al2O3) nanoparticles on root growth and development in Triticum aestivum. *Water Air Soil Pollut*. **226**, (2015).

[28] Roiter Y (2008). Interaction of nanoparticles with lipid membrane. Nano Lett..

[29] Wang S, Guo H, Li Y, Li X (2019). Penetration of nanoparticles across a lipid bilayer: Effects of particle stiffness and surface hydrophobicity. Nanoscale.

[30] Olenick LL (2018). Lipid corona formation from nanoparticle interactions with bilayers. Chem.

[31] Lee WM, An YJ, Yoon H, Kweon HS (2008). Toxicity and bioavailability of copper nanoparticles to the terrestrial plants mung bean (Phaseolus radiatus) and wheat (Triticum aestivum): Plant agar test for water-insoluble nanoparticles. Environ. Toxicol. Chem..

[32] Singh, D. & Kumar, A. Assessment of toxic interaction of nano zinc oxide and nano copper oxide on germination of Raphanus sativus seeds. *Environ. Monit. Assess*. **191**, (2019). 10.1007/s10661-019-7902-5 31673860

[33] Ofoe R (2023). Aluminum in plant: Benefits, toxicity and tolerance mechanisms. Front. Plant Sci..

[34] Farooq A, Khan I, Shehzad J, Hasan M, Mustafa G (2024). Proteomic insights to decipher nanoparticle uptake, translocation, and intercellular mechanisms in plants. Environ. Sci. Pollut. Res..

[35] FAO, 2016. Grassland Index. A searchable catalogue of grass and forage legumes. FAO, Rome, Italy|Feedipedia. https://www.feedipedia.org/node/24867.

[36] Rao KEP, de Wet JMJ, Brink DE, Mengesha MH (1987). Infraspecific variation and systematics of cultivated Setaria italica, foxtail millet (Poaceae). Econ. Bot..

[37] Kattamanchi V (2015). A novel review on Setaria italica. J. Comprehensive Pharm..

[38] Li P, Brutnell TP (2011). Setaria viridis and Setaria italica, model genetic systems for the Panicoid grasses. J. Exp. Bot..

[39] Diao X, Schnable J, Bennetzen JL, Li J (2014). Initiation of Setaria as a model plant. Front. Agric. Sci. Eng..

[40] Bennetzen JL (2012). Reference genome sequence of the model plant Setaria. Nat. Biotechnol..

[41] Huang P (2014). Population genetics of Setaria viridis, a new model system. Mol. Ecol..

[42] Li Y, Jia J, Wang Y, Wu S (1998). Intraspecific and interspecific variation in Setaria revealed by RAPD analysis. Genet. Resources Crop Evolut..

[43] Jia G (2015). Microsatellite variations of Elite Setaria varieties released during last six decades in China. PLoS One.

[44] Copper concentration in European Union soils—European Environment Agency. https://www.eea.europa.eu/data-and-maps/figures/copper-concentration-in-european-union-soils.

[45] Rahman MA (2018). Importance of mineral nutrition for mitigating aluminum toxicity in plants on acidic soils: Current status and opportunities. Int. J. Mol. Sci..

[46] Barreto DM, Tonietto AE, Lombardi AT (2021). Environmental concentrations of copper nanoparticles affect vital functions in Ankistrodesmus densus. Aquat. Toxicol..

[47] Feng S (2022). Ecological risk assessment of metallic nanoparticles on the marine environments: Species sensitivity distributions analysis. Front. Mar. Sci..

[48] Cook CM, Kostidou A, Vardaka E, Lanaras T (1998). Effects of copper on the growth, photosynthesis and nutrient concentrations of Phaseolus plants. Photosynthetica.

[49] Jiang W, Liu D, Liu X (2001). Effects of copper on root growth, cell division, and nucleolus of Zea mays. Biol. Plant.

[50] Aly, A. A. & Mohamed, A. A. *The Impact of Copper Ion on Growth, Thiol Compounds and Lipid Peroxidation in Two Maize Cultivars (Zea Mays L.) Grown in Vitro*. *AJCS* vol. 6 (2012).

[51] Azmat, R. & Riaz, S. The inhibition of polymerization of glucose in carbohydrate under Cu stress in Vigna radiata. (2012).

[52] Nair PMG, Chung IM (2015). Study on the correlation between copper oxide nanoparticles induced growth suppression and enhanced lignification in Indian mustard (Brassica juncea L.). Ecotoxicol. Environ. Saf..

[53] Gopalakrishnan Nair PM, Kim S-H, Chung IM (2014). Copper oxide nanoparticle toxicity in mung bean (Vigna radiata L.) seedlings: Physiological and molecular level responses of in vitro grown plants. Acta Physiol. Plant.

[54] Shaw AK (2014). Nano-CuO stress induced modulation of antioxidative defense and photosynthetic performance of Syrian barley (Hordeum vulgare L.). Environ. Exp. Bot..

[55] Shi, J. *et al.* Phytotoxicity and accumulation of copper oxide nanoparticles to the Cu-tolerant plant Elsholtzia splendens. **8**, 179–188. 10.3109/17435390.2013.766768 (2013). 10.3109/17435390.2013.766768 23311584

[56] Maity A (2018). Influence of metal nanoparticles (NPs) on germination and yield of oat (Avena sativa) and berseem (Trifolium alexandrinum). Proc. Natl. Acad. Sci. India Sect. B Biol. Sci..

[57] Shende, S., Rathod, D., Gade, A. & Rai, M. (2017) Biogenic copper nanoparticles promote the growth of pigeon pea (Cajanus cajan L.). *IET Nanobiotechnol* **11**, 773.

[58] Ananda S, Shobha G, Shashidhara KS, Mahadimane V (2019). Nano-cuprous oxide enhances seed germination and seedling growth in Lycopersicum esculentum plants. J. Drug Deliv. Therap..

[59] Bonilla-Bird NJ (2020). Effect of copper oxide nanoparticles on two varieties of sweetpotato plants. Plant Physiol. Biochem..

[60] Chee-González L, Muñoz-Sánchez JA, Racagni-Di Palma G, Hernández-Sotomayor SMT (2009). Effect of phosphate on aluminium-inhibited growth and signal transduction pathways in Coffea arabica suspension cells. J. Inorg. Biochem..

[61] Sun L (2020). Aluminium is essential for root growth and development of tea plants (Camellia sinensis). J. Integr. Plant Biol..

[62] Rodrigues AA (2017). Aluminum influence on Hancornia speciosa seedling emergence, nutrient accumulation, growth and root anatomy. Flora Morphol. Distribution Functional Ecol. Plants..

[63] Lee CW (2010). Developmental phytotoxicity of metal oxide nanoparticles to Arabidopsis thaliana. Environ. Toxicol. Chem..

[64] Chahardoli A, Karimi N, Ma X, Qalekhani F (2020). Effects of engineered aluminum and nickel oxide nanoparticles on the growth and antioxidant defense systems of Nigella arvensis L. Sci. Rep..

[65] Frazier TP, Burklew CE, Zhang B (2014). Titanium dioxide nanoparticles affect the growth and microRNA expression of tobacco (Nicotiana tabacum). Funct. Integr. Genom..

[66] Feregrino-Pérez, A. A. *et al.* Toxic Effects of Nanomaterials on Plant Cellular Mechanisms. *Nanomater. Interactions Plant Cell. Mech. Macromol. Agric. Implications*. 171–209 (2023). 10.1007/978-3-031-20878-2_7.

[67] Drazic, A., Myklebust, L. M., Ree, R. & Arnesen, T. (2016) The world of protein acetylation. *Biochimica et Biophysica Acta (BBA) Proteins Proteom*. **1864**, 1372–1401. 10.1016/j.bbapap.2016.06.007 27296530

[68] Tessarz P, Kouzarides T (2014). Histone core modifications regulating nucleosome structure and dynamics. Nat. Rev. Mol. Cell Biol..

[69] Shogren-Knaak M (2006). Histone H4–K16 acetylation controls chromatin structure and protein interactions. Science.

[70] Xing, G. *et al.* Genome-wide investigation of histone acetyltransferase gene family and its responses to biotic and abiotic stress in foxtail millet (Setaria italica [L.] P. Beauv). *BMC Plant Biol* **22**, (2022). 10.1186/s12870-022-03676-9 PMC9199193 35701737

[71] Luo M, Cheng K, Xu Y, Yang S, Wu K (2017). Plant responses to abiotic stress regulated by histone deacetylases. Front. Plant Sci..

[72] Kumar, B., Smita, K., Cumbal, L., Debut, A. & Pathak, R. N. Sonochemical synthesis of silver nanoparticles using starch: A comparison. *Bioinorg. Chem. Appl*. **2014**, (2014). 10.1155/2014/784268 PMC3920662 24587771

[73] Jayaraman A (2008). cDNA-AFLP analysis reveals differential gene expression in response to salt stress in foxtail millet (Setaria italica L.). Mol. Biotechnol..

[74] Imran M (2019). Comparative genome-wide analysis and expression profiling of histone acetyltransferase (HAT) gene family in response to hormonal applications, metal and abiotic stresses in cotton. Int. J. Mol. Sci..

[75] Liu, X. *et al.* Histone acetyltransferases in rice (Oryza sativa L.): phylogenetic analysis, subcellular localization and expression. *BMC Plant Biol*. **12**, 1–17 (2012). 10.1186/1471-2229-12-145 PMC3502346 22894565

[76] Harmon AC, Gribskov M, Harper JF (2000). CDPKs—A kinase for every Ca2+ signal?. Trends Plant Sci..

[77] Harmon AC, Yoo BC, McCaffery C (1994). Pseudosubstrate inhibition of CDPK, a protein kinase with a calmodulin-like domain. Biochemistry.

[78] Liese, A. & Romeis, T. Biochemical regulation of in vivo function of plant calcium-dependent protein kinases (CDPK). *Biochimica et Biophysica Acta (BBA) Mol. Cell Res*. **1833**, 1582–1589 (2013). 10.1016/j.bbamcr.2012.10.024 23123193

[79] Yu TF (2018). Genome-wide analysis of CDPK family in foxtail millet and determination of SiCDPK24 functions in drought stress. Front. Plant Sci..

[80] Saijo Y (2001). A Ca2+-dependent protein kinase that endows rice plants with cold- and salt-stress tolerance functions in vascular bundles. Plant Cell Physiol..

[81] Liu Y (2018). The calcium-dependent kinase OsCPK24 functions in cold stress responses in rice. J. Integr. Plant Biol..

[82] Ray, S., Agarwal, P., Arora, R., Kapoor, S. & Tyagi, A. K. Expression analysis of calcium-dependent protein kinase gene family during reproductive development and abiotic stress conditions in rice (Oryza sativa L. ssp. indica). *Mol. Genet. Genom*. **278**, 493–505 (2007). 10.1007/s00438-007-0267-4 17636330

[83] Dubrovina AS, Kiselev KV, Khristenko VS (2013). Expression of calcium-dependent protein kinase (CDPK) genes under abiotic stress conditions in wild-growing grapevine Vitis amurensis. J. Plant Physiol..

[84] Kong X (2013). Genome-wide identification and expression analysis of calcium-dependent protein kinase in maize. BMC Genom..

[85] Vivek, P. J., Tuteja, N. & Soniya, E. V. CDPK1 from ginger promotes salinity and drought stress tolerance without yield penalty by improving growth and photosynthesis in Nicotiana tabacum. *PLoS One* **8**, (2013). 10.1371/journal.pone.0076392 PMC3806807 24194837

[86] Dong H (2020). Overexpression of MdCPK1a gene, a calcium dependent protein kinase in apple, increase tobacco cold tolerance via scavenging ROS accumulation. PLoS One.

[87] Hutin C (2003). Early light-induced proteins protect Arabidopsis from photooxidative stress. Proc. Natl. Acad. Sci..

[88] Adamska I (1997). ELIPs—Light-induced stress proteins. Physiol. Plant.

[89] Bruno AK, Wetzel CM (2004). The early light-inducible protein (ELIP) gene is expressed during the chloroplast-to-chromoplast transition in ripening tomato fruit. J. Exp. Bot..

[90] Montané MH, Dreyer S, Triantaphylides C, Kloppstech K (1997). Early light-inducible proteins during long-term acclimation of barley to photooxidative stress caused by light and cold: High level of accumulation by posttranscriptional regulation. Planta..

[91] Valledor L, Cañal MJ, Pascual J, Rodríguez R, Meijón M (2012). Early induced protein 1 (PrELIP1) and other photosynthetic, stress and epigenetic regulation genes are involved in Pinus radiata D. don UV-B radiation response. Physiol. Plant.

[92] Zhuo C, Cai J, Guo Z (2013). Overexpression of early light-induced protein (ELIP) gene from Medicago sativa ssp. falcata Increases tolerance to abiotic stresses. Agron. J..

[93] Van Buren R, Pardo J, Wai CM, Evans S, Bartels D (2019). Massive tandem proliferation of ELIPs supports convergent evolution of desiccation tolerance across land plants. Plant Physiol..

[94] Alamillo JM, Bartels D (2001). Effects of desiccation on photosynthesis pigments and the ELIP-like dsp 22 protein complexes in the resurrection plant Craterostigma plantagineum. Plant Sci..

[95] Adamska, I., Vii, V. I., Viii, I., Aro, E.-M. & Andersson, B. The Elip family of stress proteins in the thylakoid membranes of pro- and Eukaryota. 487–505 (2001). 10.1007/0-306-48148-0_28.

[96] Panne D (2018). Mechanistic insights into histone deposition and nucleosome assembly by the chromatin assembly factor-1. Nucleic Acids Res..

[97] Zhu Y, Dong A, Shen WH (2013). Histone variants and chromatin assembly in plant abiotic stress responses. Biochim. Biophys. Acta.

[98] Dong A (2003). Regulation of biosynthesis and intracellular localization of rice and tobacco homologues of nucleosome assembly protein 1. Planta..

[99] Zhu Y, Dong A, Shen WH (2007). Chromatin remodeling in arabidopsis root growth. Plant Signal Behav..

[100] Buszewicz D (2016). HD2C histone deacetylase and a SWI/SNF chromatin remodelling complex interact and both are involved in mediating the heat stress response in Arabidopsis. Plant Cell Environ..

[101] Yung WS, Li MW, Sze CC, Wang Q, Lam HM (2021). Histone modifications and chromatin remodelling in plants in response to salt stress. Physiol. Plant.

[102] Tang L, Nogales E, Ciferri C (2010). Structure and function of SWI/SNF chromatin remodeling complexes and mechanistic implications for transcription. Prog. Biophys. Mol. Biol..

[103] Torres ES, Deal RB (2019). The histone variant H2A.Z and chromatin remodeler BRAHMA act coordinately and antagonistically to regulate transcription and nucleosome dynamics in Arabidopsis. Plant J.

[104] Hayat S (2012). Role of proline under changing environments: A review. Plant Signal Behav..

[105] Hur J, Jung KH, Lee CH, An G (2004). Stress-inducible OsP5CS2 gene is essential for salt and cold tolerance in rice. Plant Sci..

[106] Verbruggen N, Villarroel R, Van Montagu M (1993). Osmoregulation of a pyrroline-5-carboxylate reductase gene in arabidopsis thaliana. Plant Physiol..

[107] Chen C, Cui X, Zhang P, Wang Z, Zhang J (2021). Expression of the pyrroline-5-carboxylate reductase (P5CR) gene from the wild grapevine Vitis yeshanensis promotes drought resistance in transgenic Arabidopsis. Plant Physiol. Biochem..

[108] Ma L (2008). Isolation, expression analysis and chromosomal location of P5CR gene in common wheat (Triticum aestivum L.). S. Afr. J. Bot..

[109] Ghosh UK, Islam MN, Siddiqui MN, Cao X, Khan MAR (2022). Proline, a multifaceted signalling molecule in plant responses to abiotic stress: Understanding the physiological mechanisms. Plant Biol..

[110] Caburatan L, Park J (2021). Differential expression, tissue-specific distribution, and posttranslational controls of phosphoenolpyruvate carboxylase. Plants.

[111] Savouré A (1995). Isolation, characterization, and chromosomal location of a gene encoding the delta 1-pyrroline-5-carboxylate synthetase in Arabidopsis thaliana. FEBS Lett..

[112] Su M (2011). Cloning two P5CS genes from bioenergy sorghum and their expression profiles under abiotic stresses and MeJA treatment. Plant Sci..

[113] Tripathi BN, Singh V, Ezaki B, Sharma V, Gaur JP (2013). Mechanism of Cu- and Cd-induced proline hyperaccumulation in Triticum aestivum (Wheat). J. Plant Growth Regul..

[114] Klsa D (2019). Responses of phytochelatin and proline-related genes expression associated with heavy metal stress in Solanum lycopersicum. Acta Bot. Croat..

[115] Rao ASVC, Reddy AR (2008). Glutathione reductase: A putative redox regulatory system in plant cells. Sulfur Assimilation Abiotic Stress Plants..

[116] Gill SS (2013). Glutathione and glutathione reductase: a boon in disguise for plant abiotic stress defense operations. Plant Physiol. Biochem..

[117] Barna B, Gémes K, Domoki M, Bernula D, Ferenc G, Bálint B, Nagy I, Fehér A (2018). Arabidopsis NAP-related proteins (NRPs) contribute to the coordination of plant growth, developmental rate, and age-related pathogen resistance under short days. Plant Sci..

[118] Li, Y. *et al.* BcGR1.1, a cytoplasmic localized glutathione reductase, enhanced tolerance to copper stress in Arabidopsis thaliana. *Antioxidants (Basel)* **11**, (2022). 10.3390/antiox11020389 PMC8869148 35204271

[119] Yin L (2017). High level of reduced glutathione contributes to detoxification of lipid peroxide-derived reactive carbonyl species in transgenic Arabidopsis overexpressing glutathione reductase under aluminum stress. Physiol. Plant.

[120] Pilon-Smits, E. A. H., Yong Liang Zhu, Sears, T. & Terry, N. Overexpression of glutathione reductase in Brassica juncea: Effects on cadmium accumulation and tolerance. *Physiol. Plant*. **110**, 455–460 (2000) 10.1104/pp.119.1.73 PMC32244 9880348

[121] Stevens RG, Creissen GP, Mullineaux PM (1997). Cloning and characterisation of a cytosolic glutathione reductase cDNA from pea (Pisum sativum L.) and its expression in response to stress. Plant Mol. Biol..

[122] Kaminaka H, Morita S, Nakajima M, Masumura T, Tanaka K (1998). Gene cloning and expression of cytosolic glutathione reductase in rice (Oryza sativa L.). Plant Cell Physiol..

[123] Contour-Ansel D, Torres-Franklin ML, Cruz De Carvalho MH, D’Arcy-Lameta A, Zuily-Fodil Y (2006). Glutathione reductase in leaves of cowpea: Cloning of two cDNAs, expression and enzymatic activity under progressive drought stress, desiccation and abscisic acid treatment. Ann. Bot..

[124] Guerinot, M. Lou. The ZIP family of metal transporters. *Biochimica et Biophysica Acta (BBA) Biomembranes* **1465**, 190–198 (2000). 10.1016/s0005-2736(00)00138-3 10748254

[125] Ajeesh Krishna TP, Maharajan T, Victor Roch G, Ignacimuthu S, Antony Ceasar S (2020). Structure, function, regulation and phylogenetic relationship of ZIP family transporters of plants. Front. Plant Sci..

[126] Milner MJ, Seamon J, Craft E, Kochian LV (2013). Transport properties of members of the ZIP family in plants and their role in Zn and Mn homeostasis. J. Exp. Bot..

[127] Li XW, Liu HJ, Xie SX, Yuan HY (2013). Isolation and characterization of two genes of the early light-induced proteins of Camellia sinensis. Photosynthetica.

[128] Xu L (2015). De novo sequencing of root transcriptome reveals complex cadmium-responsive regulatory networks in radish (Raphanus sativus L.). Plant Sci..

[129] Ramesh SA, Shin R, Eide DJ, Schachtman DP (2003). Differential metal selectivity and gene expression of two zinc transporters from rice. Plant Physiol..

[130] Durmaz E (2011). Expression and cellular localization of ZIP1 transporter under zinc deficiency in wild emmer wheat. Plant Mol. Biol. Rep..

[131] Wang, T. *et al.* (2020) Transcriptome analysis provides insights into grain filling in foxtail millet (Setaria italica L.). *Int. J. Mol. Sci*. **21**, 5031. 10.3390/ijms21145031 PMC7403974 32708737

[132] Nguyen DQ, Eamens AL, Grof CPL (2018). Reference gene identification for reliable normalisation of quantitative RT-PCR data in Setaria viridis. Plant Methods.

[133] Kumar K, Muthamilarasan M, Prasad M (2013). Reference genes for quantitative real-time PCR analysis in the model plant foxtail millet (Setaria italica L.) subjected to abiotic stress conditions. Plant Cell Tissue Organ Cult..

[134] Qin L (2020). Genome-wide gene expression profiles analysis reveal novel insights into drought stress in foxtail millet (Setaria italica L.). Int. J. Mol. Sci..

