# Supplementary material for: Effects of copper, and aluminium in ionic, and nanoparticulate form on growth rate and gene expression of Setaria italica seedlings
Source: Sci Rep. 2024 Jul 10;14:15897. doi: 10.1038/s41598-024-66921-1 (PMC11237061; doi:10.1038/s41598-024-66921-1)
Supplement: Supplementary file 1 — Supplementary Information. [file 41598_2024_66921_MOESM1_ESM.docx]

**Table.** Starters used in real-time analysis.

| Gene | Forward primer | Reverse primer | Tm | Reference |
| --- | --- | --- | --- | --- |
| α-ACT | 5’-cgcatatgtggcttctgact-3’ | 5’ggggcacctaaatctctctgc-3’ | 55°C | ^1^ |
| β-TUB | 5’-taccagccaccatctgttgt-3’ | 5’-ggtcgaacttgtggtcaatg-5’ | 56°C | ^2^ |
| ACT-1 | 5’-gcagcgggtcatggattc-3’ | 5’-agcgagcagcggtagacaa-3’ | 59°C | ^2^ |
| CDPK | 5’-cagaattgacagagaatgaaatcca-3’ | 5’-gatggttccgctgttgtcaata-5’ | 58°C | ^2^ |
| LIP | 5’-gccaagataggaatccattgct-3’ | 5’-cctcggatctgtcacgagtaga-3’ | 68°C | ^2^ |
| NFC | 5’-gctgcactccgaggaactg-3’ | 5’-cacggagctttcaacggatt-3’ | 58.6°C | ^2^ |
| P5CR | 5’-gttggacggctaggtgtgaa-3’ | 5’-ctgtatgccacctgattctgc-3’ | 60°C | ^1^ |
| P5CS | 5’-gtcatcagaggagcgtaa-3’ | 5’-gttatctttcctggcttta-3’ | 58.5°C | ^1^ |
| GR | 5’-tggacgtccgggctcaca-3’ | 5’-tcggctgctgaagagtctgtaa-3’ | 45°C | ^2^ |
| SiZIP1 | 5’-caacctcttcgtcatcgtcaag-3’ | 5’-ctcttccggttgtagaaggtgag-3’ | 57.3°C | ^3^ |

**References**

1. Qin, L. *et al.* Genome-wide gene expression profiles analysis reveal novel insights into drought stress in foxtail millet (*Setaria italica* L.). *Int J Mol Sci* **21**, 1–21 (2020).
2. Jayaraman, A. *et al.* cDNA-AFLP analysis reveals differential gene expression in response to salt stress in foxtail millet (*Setaria italica* L.). *Mol Biotechnol* **40**, 241–251 (2008).
3. Alagarasan, G., Dubey, M., Aswathy, K. S. & Chandel, G. Genome wide identification of orthologous ZIP genes associated with zinc and iron translocation in Setaria italica. *Front Plant Sci* **8**, (2017).
